# Application of Morphological Segmentation to Leaking Defect Detection in Sewer Pipelines

**DOI:** 10.3390/s140508686

**Published:** 2014-05-16

**Authors:** Tung-Ching Su, Ming-Der Yang

**Affiliations:** 1 Department of Civil Engineering and Engineering Management, National Quemoy University, Da Xue Rd. 1, Kinmen 892, Taiwan; E-Mail: spcyj@nqu.edu.tw; 2 Department of Civil Engineering, National Chung Hsing University, Taichung 402, Taiwan

**Keywords:** leaking, sewer pipeline, computer vision, defect detection, morphology

## Abstract

As one of major underground pipelines, sewerage is an important infrastructure in any modern city. The most common problem occurring in sewerage is leaking, whose position and failure level is typically idengified through closed circuit television (CCTV) inspection in order to facilitate rehabilitation process. This paper proposes a novel method of computer vision, morphological segmentation based on edge detection (MSED), to assist inspectors in detecting pipeline defects in CCTV inspection images. In addition to MSED, other mathematical morphology-based image segmentation methods, including opening top-hat operation (OTHO) and closing bottom-hat operation (CBHO), were also applied to the defect detection in vitrified clay sewer pipelines. The CCTV inspection images of the sewer system in the 9th district, Taichung City, Taiwan were selected as the experimental materials. The segmentation results demonstrate that MSED and OTHO are useful for the detection of cracks and open joints, respectively, which are the typical leakage defects found in sewer pipelines.

## 1. Introduction

Sewerage is an important infrastructure in modern cities so the pipeline authorities in Taiwan have been making considerable efforts to construct sewer systems. Nowadays, the percentage of sewer system coverage in Taiwan is reported to be 32.9% [1]. The most common problem occurring in sewer pipelines is leakage, whose position and failure level can be idengified through image inspection in order to facilitate the rehabilitation process. Distributing fiber optics is an efficient way to detect and localize leakages along pipelines by monitoring pressure or temperature differences. Fiber optic systems have been demonstrated useful in monitoring leakage of buried pipelines, such as oil and gas pipelines [2–5], but sewerage consists of gravity pipelines rather than pressure pipelines so fiber optics are inapplicable for leakage detection of sewerage.

Sewer rehabilitation involving sewer inspection, assessment of structural conditions, computation of structural condition grades, and determination of rehabilitation methods and substitution materials is necessary for the buried sewer pipes to maintain the designed drainage capability [6–10]. At present, closed circuit television (CCTV) is the most popular equipment for sewer inspection because of its low cost compared with sewer scanner evaluation technology (SSET) cameras, ground piercing radar (GPR), sonar, and infrared thermograph [11–14]. However, human fatigue and subjectivity, and time-consumption for engineers may be barriers for detecting and diagnosing defects in sewer pipelines via CCTV images due to the great number of images that must be inspected. Recently, image processing and argificial intelligence techniques were applied as diagnostic systems to assist engineers in interpreting sewer pipe defects in inspection images [15–20].

Among the image processing techniques, mathematical morphology-based image segmentation has been extensively applied in pattern recognition research [18–25]. Erosion and dilation are two basic operators of morphological segmentation, and are usually operated in tandem for the image enhancement of objects of interest [26]. During the erosion and dilation operations, the computation of mathematical logic, including intersection and union, is simultaneously introduced into the morphology analysis. A determination of structural element (SE) is necessary before the operation of erosion and dilation. The size and shape of SE depend on the objects of interest. For example, linear SE was adopted in the operation of erosion and dilation for segmenting cracks from CCTV images [15].

Erosion and dilation with linear structural elements were performed to segment vessel-like patterns, which are very common in medical images [21]. Then, a cross-curvature evaluation was implemented to differentiate vessels from analogous background patterns. Mathematical morphology and curvature evaluation were also employed in detecting crack patterns in pipeline images [18]. Sinha and Fieguth used opening top-hat operation (OTHO), in which firstly erosion followed by dilation (called image opening) was applied to an SSET inspection image, secondly the opening operated image was subtracted from its SSET inspection image (called top-hat operation), and finally the top-hat operated image was transferred into a binary one by Otsu technique to segment pipeline defects, including cracks, holes/joints, laterals, and pipe collapse, in sewer pipelines [19]. However, environmental noise or poor image quality would deteriorate the performance of OTHO so as to hamper the pipeline defect detection. To effectively and correctly detect sewer pipeline defects, this paper presents a novel algorithm, morphological segmentation based on edge detection (MSED), to segment sewer pipeline defects from CCTV inspection images. Edge detection is considered as an important pre-processing step in image segmentation [27–29]. Based on edge detection, MSED attempts to search complete and correct image regions of sewer pipeline defects in CCTV images.

## 2. Methodology

**Figure 1.**
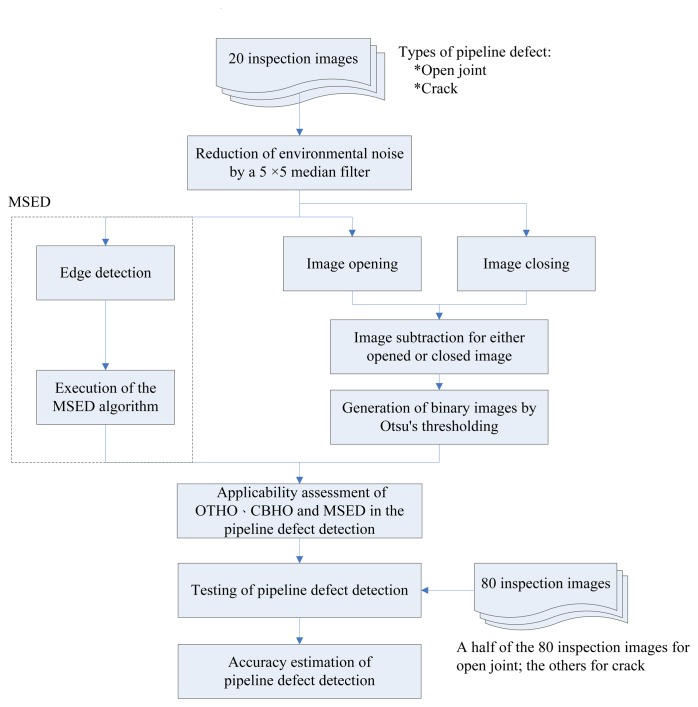
Research scheme.

In addition to OTHO, closing bottom-hat operation (CBHO), which is a dual operation of OTHO, was also employed to detect pipeline defects in this research. Figure 1 shows the schematic outline of this research. Edge detection and MSED algorithm are involved in the MSED method, whereas image opening, image closing, image subtraction, and Otsu's thresholding are involved in the OTHO or CBHO methods. During sewer inspection, both pipeline defects and environmental noise are imaged by the CCTV recorder so complete and correct segmentation of pipeline defects from the CCTV inspection images becomes difficult [10]. Environmental noises, such as shadows and stains resulting from inappropriate imaging condition of the CCTV robot and aged pipes, respectively, are different from image noises, such as impulsive noise, Gaussian noise, speckle noise, and periodic noise. However, sometimes stains inside pipeline shows an analogy of impulsive noise in CCTV images. Median filter, one of the smoothing filters, is well suited to remove impulsive noise [30]. Before the operation of OTHO, CBHO or MSED, a median filtering of a 5 × 5 window is adopted to reduce rather than remove the impact of the environmental noises on the image segmentation. The performance of the environmental noise reduction by OTHO and MSED had been demonstrated in the literature of Su *et al.* [10], who indicated that MSED outperforms OTHO. Nevertheless, the correctness and completeness of the pipeline defect segmentation by OTHO and MSED have not been estimated. After the image segmentation by OTHO, CBHO or MSED, the accuracy of the pipeline defect detection was estimated by comparing the segmentation result with the manual interpretation data.

### 2.1. Median Filtering

Digital numbers of pixels possess the characteristic of signal variance in a two-dimensional space. If significant differences appear between the digital numbers, the image details, including objects and noise, can be detected by proper image processing techniques. Low-pass filters, including mean filter, Gaussian filter, and median filter, can be applied for noise removal [30–32]. Regarded as an image smoother, low-pass filtering replaces each digital number in an input image by the mean of its neighbors and itself. Mean filtering has the capability of effectively removing signals with great variance, but sometimes a median filter outperforms a mean filter in the sense of preserving useful image details [30]. In this paper, a median filter was applied to the inspected images before image segmentation. The size and shape of a kernel must be determined prior to median filtering. A square kernel consisting of *M* × *M* elements is usually applied to median filtering. Generally, *M* must be an odd number above or equal to 3 (*M* = 5 was adopted in this paper) so that kernel has the only central element to be filtered [33].

### 2.2. Opening Top-Hat Operation (OTHO)

Image segmentation can be performed by the OTHO method to produce a binary image, in which the image regions consists of white pixels expressing the objects of interest, *i.e.*, pipeline defects in this research, and black pixels denoting the background environment. Considering a structuring element as a parameter to morphological operation, the light and dark portions in an image can be reshaped or morphed in various ways [19], such dilation and erosion as two basic morphological operations [34]. Sets **M** and **S** represent a median filtered image consisting of pixels *p*(*x*,*y*) and a structuring element, respectively:

(1) M={(x,y)|p(x,y)}

(2) S={(x,y)|(x,y)in structuring element}

The dilation of **M** by **S**, denoted **M**⊕**S**, is the union of all pixels in **M** surrounded by the shape of **S** and defined as:

(3) M⊕S={m+s|for allm∈Mands∈S}

Similarly, the erosion of **M** by **S**, denoted **M**Θ**S**, removes all pixels within a “distance” **S** from the edge of **M** and is defined as:

(4) MΘS={m|s+m∈Mfor everys∈S}

Based on Equations (3) and (4), the opening operation is defined as:

(5) M∘S=(MΘS)⊕S

Image regions rarely relative to the structuring element are removed by the opening operation while preserving image regions greater than structuring elements [19]. Based on Equation (5), OTHO is defined as:

(6) $${\text{OTHO}}_{M\circ S}=M-(M\Theta S)⊕S$$

Equation (6) can be regarded as an opening operated image extracted from the median filtered one, and is also called image subtraction or top-hat operation [32]. Based on Equation (1) through Equation (6), the structural element **S**, which is a matrix consisting of only 0s and 1s, has a great impact on the performance of OTHO. In the matrix, the eight-neighbor pixels of 1s can be assigned as of arbitrary shape and size. Diamond, disk, line, rectangular, and square are the common structuring elements; nevertheless, line structuring elements are not frequently adapted due to its ability of detecting a single border [35]. In inspection images, open joints looks like illuminated ring-like objects, but cracks usually present an irregular or linear pattern. Due to the great difference between the patterns of open joints and cracks, a disk SE with a radius of 3 pixels was adopted to assist OTHO in morphologically probing the pipeline defects.

### 2.3. Closing Bottom-Hat Operation (CBHO)

CBHO is the dual operation of OTHO. Image dilation and erosion are two basic operations of image opening and closing, whereas image closing is an inverted operation of image opening and defined as:

(7) M•S=(M⊕S)ΘS

Based on Equation (7), CBHO is defined as:

(8) $${\text{CBHO}}_{M•S}=(M⊕S)\Theta S-M$$

### 2.4. Morphological Segmentation Based on Edge Detection (MSED)

Another image segmentation method, MSED, was employed to segment the pipeline defects from inspection images. Based on edge detection results, the MSED algorithm attempts to transform black pixels between two detected edges into white pixels. Edge detection technique and the MSED algorithm are introduced as follows.

#### 2.4.1. Edge Detection

Edge detection is the most common approach for detecting meaningful discontinuities in gray level [36]. Edge detector, *i.e.*, a high-pass filter, allows high-frequency components but excludes low-frequency ones to pass [30]. In this research, the Sobel first derivative edge detector was taken into consideration for the edge detection of the pipeline defects in the median filtered image **M**.

#### 2.4.2. The MSED Algorithm

A hybrid approach, integrating mathematical morphological edge detection, contour-based image segmentation, and trajectory estimation, was presented to track multiple objects in video frames [26]. Region growing and merging were introduced into the contour-based image segmentation algorithm, so several initial growing seeds are needed for searching the morphologies of the contoured objects. In the MSED algorithm, firstly edge detection generates a binary image (or logical image), in which 1 s and 0 s represent the border of pipeline defects and background environment, respectively. Secondly, the MSED algorithm applies region growing to the morphological segmentation of pipeline defect based on the binary image so that initial growing seeds are unneeded. A brief illustration of the MSED algorithm can be referred to the literature of Su *et al.* [10], and is encoded in Matlab as follows. In the following operation, *d_lim_* is used to survey the minimum distance between two border pixels in either row or column direction. Once the minimum distance is determined, the interval pixels (background pixels) are defined as the pipeline defect pixels:
For *d* = 2:*d_lim_* % *d_lim_ is an integer, and d* = *2, 3, 4*, …, *d_lim_ are tested*. For *i* = 1:*m* % *m and n denote the pixel number of the image region in the row and col*.   For *j* = 1:*n* − *d*     If (image (*i*,*j*) = = 1 & image (*i*,*j* + *d*) = = 1) % *image (i,j) is edge detection binary image*.       image (*i*,*j* + 1:*j* + *d* − 1) = 1;     End   EndEndFor *j* = 1:*n* For *i* = 1:*m* − *d*   If (image (*i*,*j*) = =1 & image (*i* + *d*,*j*) = =1)     image (*i* + 1:*i* + *d* – 1,*j*) = 1;   End   End EndEnd

### 2.5. Accuracy Estimation of Pipeline Defect Detection

To verify the three segmentation methods in pipeline defect detection, the accuracy indices, including completeness (*Compl*), correctness (*Corr*), and quality, are introduced as [18]:

(9) $$\text{compl}=(S_{e}\cap M_{s})/M_{s},$$

(10) $$\text{Corr}=(S_{e}\cap M_{s})/S_{e},$$

(11) Quality=(Compl*Corr)/(Compl−Compl*Corr+Corr),

where *S_e_* is the segmentation by OTHO, CBHO, or MSED methods; *M_s_* expresses the image regions of true pipeline defects interpreted by experts *a priori*.

## 3. Results and Discussion

**Figure 2.**
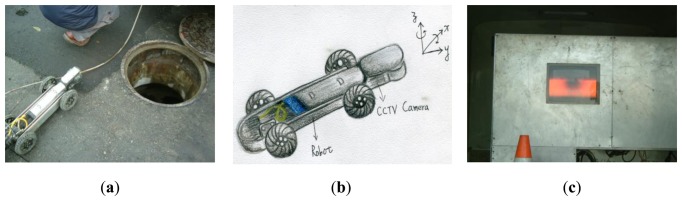
(**a**) CCTV robot. (**b**) Illustration of the CCTV camera rotating along the *x* and *z* axes. (**c**) The monitoring system of the CCTV robot.

The vitrified clay sewer pipelines in Taichung City, which is the biggest city in central Taiwan, were inspected by a CCTV camera mounted on a robot. The video streams are in MPEG at 30 frames per second. A manhole to manhole distance was scheduled as an inspection unit and required about 10 min for CCTV shooting. Thousands frames of inspection images were recorded in each video stream. Figure 2 shows the CCTV robot and its corresponding monitoring system inside the vehicle. The CCTV robot is connected with the vehicle on the ground by a power cable. The power cable provides two utilities for the inspection task, including delivering image signal back to the vehicle and pulling the CCTV robot back while the inspection task of a pipeline length (a distance between two manholes) is finished. Moreover, the CCTV camera can be rotated along *x* and *z* axes if necessary (see Figure 2b). This paper used the software_VirtualDub, which can rank images in a row according to imaged time, to capture 100 frames of inspection images from the video streams as experimental materials, and assessed the applicability of the three segmentation methods for pipeline defect detection.

The inspection results show that cracks and open joints are two typical pipeline defects causing leakage problems in the sewer system. Due to the dark environment inside sewerage, a light beam from the CCTV robot is needed during CCTV inspection. The optical axis of CCTV robot would be usually parallel to the central axis of pipeline during sewer inspection. Consequently, open joints are apt to be illuminated by the CCTV robot because the surface of an open joint is usually perpendicular to the central axis of the pipeline. Cracks in CCTV images usually can be regarded as clearly dark patterns [14], but sometimes appear as bright patterns under several specific imaging conditions.

To demonstrate the performance of OTHO, CBHO, and MSED in the pipeline defect detection, 20 inspection images, in which each of cracks and open joints were individually recorded in 10 inspection images, were offered for training the three segmentation methods. The applicability of the proposed segmentation methods in the detection of cracks and open joints was also discussed. Then, other 40 inspection images recording either cracks or open joints were used for testing the proposed segmentation methods.

### 3.1. Applicability Assessment of OTHO, CBHO and MSED in Pipeline Defect Detection

**Figure 3.**
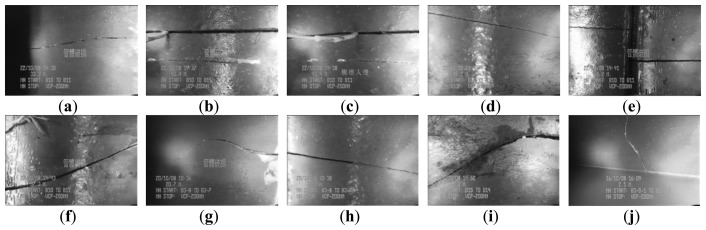
CCTV images of cracks.

**Figure 4.**
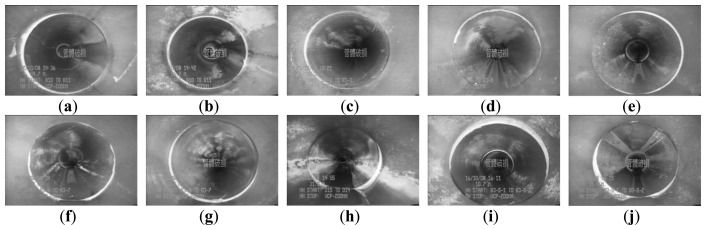
CCTV images of open joints.

**Figure 5.**
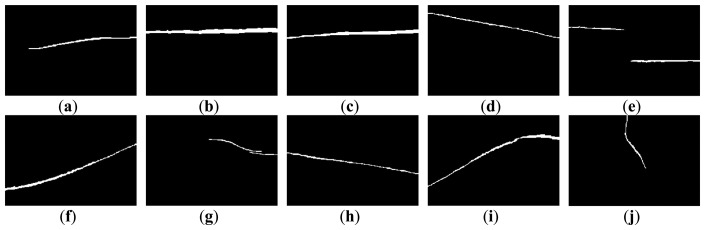
Manual interpretation of cracks corresponding to images in Figure 3.

**Figure 6.**
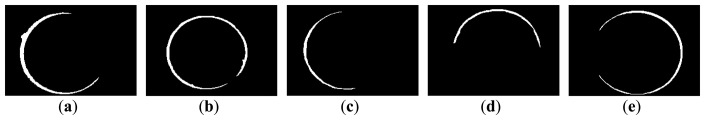

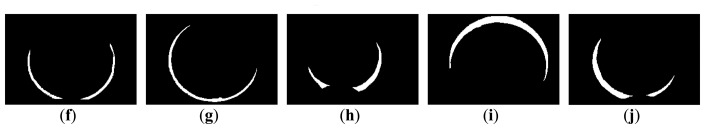
Manual interpretation of open joints corresponding to images in Figure 4.

Figures 3 and 4 show 10 inspection images of cracks and open joints, respectively, used for assessing the applicability of OTHO, CBHO, and MSED in pipeline defect detection. To reduce the computation, this research detected pipeline defects by probing the discontinuities in gray level instead of color imagery. The human interpretation of the pipeline defects in Figures 3 and 4 is shown in Figures 5 and 6, respectively. The white image regions express the interpreted pipeline defect, whereas the black ones represent the background environment.

#### 3.1.1. Pipeline Defect Detection Using OTHO

**Figure 7.**
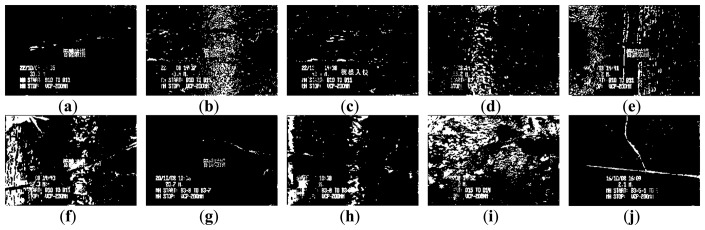
Segmentation of crack CCTV images by OTHO.

**Figure 8.**
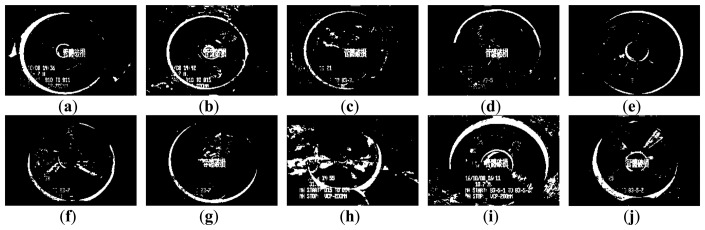
Segmentation of open joint CCTV images by OTHO.

Figures 7 and 8 are the segmentation applying OTHO to the images in Figures 3 and 4, respectively. Based on Equation (9) through Equation (11), Table 1 lists the segmentation accuracy in Figures 7 and 8, respectively, and shows that OTHO is better in open joint detection than crack detection. In Figure 4, open joints look like ring patterns with a strong illumination, whereas most cracks present slender gap patterns without illumination in Figure 3. Figures 7 and 8 show that pipeline defects or noisy environments with stronger illumination than their neighbors were detected by OTHO as white image regions.

In Table 1, OTHO offering an averaged *Compl* of 89.90% coupled with a standard deviation of 4.32% for open joint detection, indicating that most image regions belonging to open joints can be well detected. However, not only the open joints but also the inspection texts or the environmental noise were detected so much the *Corr* is far inferior to the *Compl*. The inspection texts, including date, manholes of departure and arrival, pipe material, diameters, and category of pipe defect, were recoded and placed on the inspection image. If the tone of the inspection texts against their neighbor background is clear, the inspection texts have a high probability of being segmented and decrease the *Corr*. Thus, in the future a separate attribute database to record the inspection texts is necessary to avoid incorrect detections of pipeline defects. In addition to the inspection texts, we found that the heterogeneous illumination due to the reflection by the noisy environments would also result in the incorrect detection of pipeline defects and worsened *Quality*.

Table 1 also shows that applying OTHO to the open joint detection in Figure 4e obtained the best *Quality* of 67.64%, where *Compl* and *Corr* are 88.16% and 74.39%, respectively. Figure 8e shows that OTHO has the capability of detecting the near open joint (larger white ring) and the far open joint (smaller white ring) from the CCTV position. However, the near open joint referred to in Figure 6e should be the only target of concern under the assumption of only one defect existing in one image in this research. The *Corr* of 74.39% can be improved by ignoring the far open joint.

As for the crack detection by OTHO, the best *Compl* of 71.60% was obtained for the crack in Figure 3j due to its strong illumination. However, the transverse stripe of non-crack and the inspection texts in Figure 3j were also detected so that the *Corr* of merely 8.64% indicates that OTHO is unsuitable for crack detection.

#### 3.1.2. Pipeline Defect Detection Using CBHO

**Figure 9.**
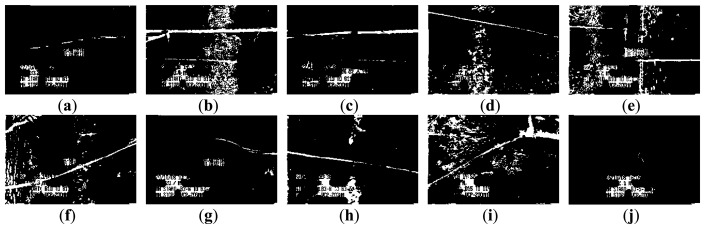
Segmentation of crack CCTV images by CBHO.

**Figure 10.**
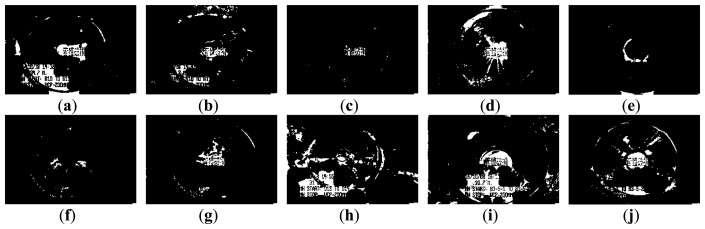
Segmentation of open joint CCTV images by CBHO.

The segmentation applying CBHO to the corresponding to images in Figures 3 and 4 are shown in Figures 9 and 10, respectively. The accuracy of the detected pipeline defects in Figures 9 and 10 is estimated in Table 2. Obviously, CBHO derives better performance in detecting cracks than open joints, contrary to OTHO. Comparing Tables 1 and 2, *Quality* of crack detection is significantly improved and demonstrates that CBHO is suitable for detecting objects which absorb light beams. Excluding Figure 9a and 9j, most segmented cracks in Figure 9 have *Compl* above 50% by CBHO. In particular, the crack in Figure 9e obtained a *Compl* of 82.79%. Also, the inspection text or environmental noise was detected by CBHO so that the crack in Figure 9e has *Corr* of 8.94% which is far inferior to *Compl* of 82.79%. Consequently, the low *Corr* results in low *Quality* of 8.78%.

Table 2 also shows that CBHO is unsuitable for open joint detection due to low *Quality*. Even, image (e) was given the *Quality* of 0% with *Compl* and *Corr* both being 0%. Figure 10e is seen that the farther open joint in Figure 4e was segmented as the ring-like image region, but the farther open joint is not the detected defect shown as Figure 6e. In addition to the ring-like segmentation, the other segmented image region is located at the bottom of Figure 10e. Unfortunately, the segmented image regions in Figure 10e do not match the manual interpretation in Figure 6e at all. The above result illustrates that CBHO would lead to pseudosegmentation for open joints.

#### 3.1.3. Pipeline Defect Detection Using MSED

**Figure 11.**
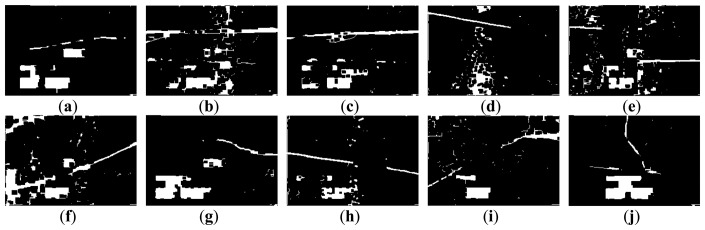
Segmentation of crack CCTV images by MSED.

**Figure 12.**
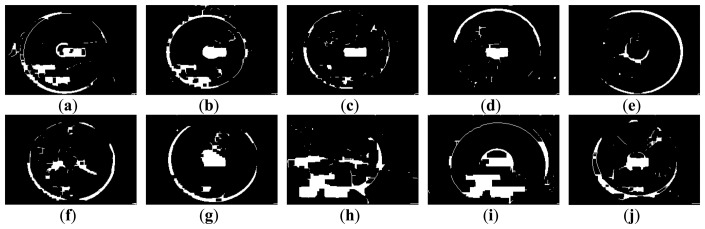
Segmentation of open joint CCTV images by MSED.

Figures 11 and 12 show the segmentation applying MSED to Figures 3 and 4, respectively. Table 3 presents the segmentation accuracy for Figures 11 and 12, and shows that MSED offers better performance for open joint detection than crack detection. Moreover, a comparison between Tables 2 and 3 shows MSED is superior to CBHO in crack detection due to the less segmentation of environmental noise. However, the inspection texts were also detected by MSED as white rectangles (see Figures 11 and 12), and this greatly deteriorated the segmentation. In Table 3, the best *Quality* of 26.51% with *Compl* of 80.29% and *Corr* of 28.36% was obtained for the crack detection in Figure 3c. Especially, the *Corr* of 28.36% can be greatly improved if the inspection texts were removed from the image. Similarly, in Table 3 lower *Corr* values, such as 7.60%, 5.11% or 5.12%, resulted from the detection of the inspection texts so as to deteriorate the *Quality* of 6.91%, 5.01% or 5.03% (see Figure 11a, g or j). Although a crack usually appears as a slender gap pattern without any illumination, comparison of Table 3 to Table 2 demonstrates that MSED has better capability of crack detection than CBHO, unless a crack is located in shadows or is smoothed by the surroundings. Noticeably, the pattern of a crack in Figure 3b is extremely visible. However, a *Compl* of merely 49.51% was obtained by MSED for the crack detection that is caused by the parameter, *d_lim_*, given by a length of 7 pixels, which is short for the MSED algorithm in complete crack detection (see Figure 11b).

In Table 3, the four segmented images of open joints, including images (d), (e), (f), and (g), gave better *Quality*. Especially for image (e), *Quality* of 54.47% with *Compl* of 90.89% and *Corr* of 57.62% was the optimum derived by MSED. As in Figures 8e and 10e, in Figure 12e the farther open joint was also detected by MSED as a smaller white ring that decreased segmentation correctness. In other words, the *Corr* of 57.62% is an underestimation for MSED in the performance of correctly detecting pipeline defects. In Table 3, the best and worst *Compl*s were obtained for the segmentation of Figure 12d and 12i, respectively. The open joints in Figure 4d and 4i have similar patterns. The greatest difference between two patterns is the illuminated areas of the open joints due to the large displacement of the open joint in Figure 4i compared with Figure 4d. Unfortunately, the upper edge of the open joint in Figure 4i failed to be detected by edge detection causing the MSED algorithm to be ineffective in the open joint detection (see Figure 12i). The failed edge detection was caused the image gradients below the threshold which was automatically determined by Otsu's technique.

#### 3.1.4. Summary of Pipeline Defect Detection Using OTHO, CBHO and MSED

On a 2.5 GHz PC with four CPUs, less than 0.1, 0.5, and 1 second was taken to process the MATLAB code of CBHO, OTHO, and MSED, respectively. With superior computation efficiency, in-time pipeline defect detection can be expected. Based on the illustration of applying OTHO, CBHO, and MSED to the detection of crack and open joint, a summary of the pipeline defect detection using OTHO, CBHO, and MSED is obtained and discussed as follows:

(1) Tables 1 and 3 show that *Quality* of open joint detection is higher than that of crack detection. Cracks were found to be more difficult to detect due to their varied appearance. The *Quality* obtained by MSED (see Table 3) is significantly better than that by OTHO for the crack detection (see Table 1), whereas OTHO is superior to MSED in open joint detection. The comparison in Tables 2 through 3 indicates that MSED seems better than CBHO in both crack and open joint detection. Hence, this paper suggests that OTHO and MSED can be considered suitable for open joint and crack detections, respectively.
(2) The difficulty to derive a high *Corr* results from the serious interference from the noisy environment which is unavoidable during sewer inspection. OTHO, offering *Compl* between 83.92% and 98.26% for the open joint detection (see Table 1), is proven useful for open joint detection. *Compl* values up to 83.30% and above 60% for seven of the 10 inspection images derived by MSED for the crack detection (see Table 3) demonstrate the effectiveness of MSED in crack detection.
(3) Figures 11 and 12 show that most inspection texts were detected by MSED as image regions consisting of white rectangles. However, the sizes of the white rectangles are mostly larger than those of the detected inspection texts by OTHO. The larger the detected inspection texts are, the more inferior the *Corr* is. In addition, the number of the image regions detected by MSED is significantly less than that by OTHO. In Figures 7 and 8, many small image regions due to the noisy environment, such as heterogeneous illumination, were detected by OTHO and this severely deteriorated *Corr*.
(4) Figures 7, 8, 9, 10, 11 and 12 show that in crack detection the three segmentation methods suffer from the deterioration caused by environmental noise. The segmentation of environmental noise results from the corroded pipe wall which reflects heterogeneous beams back to the CCTV camera. In an image with heterogeneous beam reflection, OTHO and CBHO would segment brighter and darker portions, respectively. MSED would detect the edges between the bright and dark portions in image segmentation. The segmented environmental noise frequently has the morphological feature of fractals which is extremely different from the line-like features of cracks or the ring-like features of open joints. In future work, criteria based on morphological features should be established to remove the environmental noise, and the effect of the noise removal will be discussed.

### 3.2. Verification of OTHO and MSED in Pipeline Defect Detection

**Figure 13.**
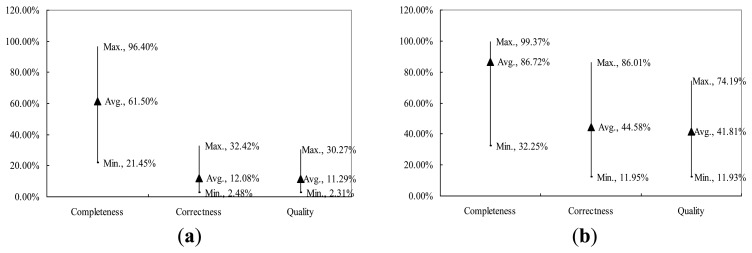
Verification of pipeline defect detection. (**a**) Crack detection using MSED. (**b**) Open joint detection using OTHO.

To verify the applicability of OTHO and MSED in open joint and crack detection, this research employed additional 40 inspection images for each of crack and open joint. Based on the total 80 inspection images, Figure 13 shows *Compl*, *Corr*, and *Quality* for the applicability verification. Figure 13 displays the accuracies of MSED and OTHO tests in the crack and open joint detections, respectively. The long bar represents a great difference between the maximum and minimum of *Compl*. Averaged *Compl* values of 61.50% and 86.72% were obtained for the crack and open joint detections, respectively. The standard deviations of *Compl* in the crack and open joint detections are 19.98% and 13.59%, respectively. In conclusion, OTHO has a robust capability of detecting open joints and MSED can effectively detect cracks.

Figure 13 also shows *Corr* of the open joint detection ranging significantly wider than that of the crack detection. The standard deviations of *Corr* for the open joint and crack detections are 18.49% and 5.89%, respectively, which is similar to that of *Quality*. Compared with Figure 13a and 13b, *Corr* is greatly inferior to *Compl* due to the influence of the noisy environment. Moreover, it is more difficult to correctly detect cracks than open joints so that *Quality* of the crack detection is relatively lower than that of the open joint detection. Table 4 is a statistical analysis of the different levels of the accuracy indices for the pipeline defect detection. For the crack detection by MSED, almost all 40 inspected images obtaining both *Corr* and *Quality* less than 20% indicates that the noisy environment has a great impact on MSED performance of pipeline defect detection. For the open joint detection using OTHO, most of 40 inspected images obtained *Compl* values above 80% and *Corr* ones between 40% and 80%. Moreover, *Corr* has greater impact on *Quality* than *Compl*.

## 4. Conclusions

This paper proposes a novel image segmentation method, morphological segmentation based on edge detection (MSED), to detect the pipeline defects, including cracks and open joints, which are the typical pipeline defects causing leaking problems in vitrified clay sewage pipelines. The developed image segmentation methods, opening top-hat operation (OTHO) and closing bottom-hat operation (CBHO), were also applied to the pipeline defect detection for comparison. Various indices, including completeness, correctness, and quality, were introduced into the accuracy assessment of the pipeline defect detection. Cracks were found to be more difficult to detect due to their varied appearance. The test results demonstrate that MSED outperforms both CBHO and OTHO in crack detection, and OTHO outperforms both CBHO and MSED in open joint detection. In conclusion, this paper suggests employing MSED for cracks and OTHO for open joints in detection processing.

The sewer pipes in the 9th district sewer system of Taichung City were made of vitrified clay, which is a typical rigid pipe material. The pipeline defect detection methods proposed in this research can also be applied to other rigid pipes, such as concrete pipes. However, the inspection texts recorded onto the acquired CCTV images deteriorate the correctness and quality. The barrier of the noisy environment segmentation for MSED or OTHO remains. We strongly suggest that a separate attribute database for inspection texts is necessary in future CCTV shooting processes. Removal of the inspection texts from CCTV images should significantly improve the detection correctness. Moreover, some morphology-based decision criteria or fitness function [37] could be established in the future for removal of the noisy environment to improve the correctness and quality of detection. According to the comment by Kirstein *et al.* [29], the irregular environment inside sewer pipelines results in a long time still being needed for developing an automated sewer inspection system. In further study, ground light detection and ranging (LiDAR) can be introduced and coupled with a synchronous camera for pipeline defect measurement based on point clouds with precise coordinates. Nevertheless, this paper demonstrates that the different types of pipeline defects need different image segmentation methods for detection. Thus, idengifying an appropriate image segmentation method for the detection of objective pipeline defects also needs advanced study.

## Figures and Tables

**Table 1.** Segmentation accuracy of crack (C) and open joint (OJ) segmentations by OTHO.

**Image ID**	**Accuracy Index (%)**					

	***Compl***		***Corr***		***Quality***	

	**C**	**OJ**	**C**	**OJ**	**C**	**OJ**
(a)	8.28	98.26	2.80	49.25	2.14	48.82
(b)	6.91	88.14	3.39	41.12	2.33	38.97
(c)	2.44	83.92	1.60	31.32	0.97	29.55
(d)	1.56	91.73	0.28	47.33	0.23	45.40
(e)	1.41	88.16	0.19	74.39	0.17	67.64
(f)	19.93	86.21	4.77	55.92	4.00	51.33
(g)	5.40	87.43	0.91	56.24	0.79	52.03
(h)	0.56	92.66	0.07	19.21	0.07	18.93
(i)	9.57	92.56	0.78	39.66	0.73	38.44
(j)	71.60	91.30	8.64	47.97	8.35	45.88
**Average**	6.23	89.90	1.64	46.05	1.27	43.46
**Standard Deviation**	6.08	4.32	1.65	15.89	1.31	14.11

p.s. The extreme values of Image (j) of crack are excluded in calculation of average and standard deviation.

**Table 2.** Segmentation accuracy of crack (C) and open joint (OJ) segmentations by CBHO.

**Image ID**	**Accuracy Index (%)**					

	***Compl***		***Corr***		***Quality***	

	**C**	**OJ**	**C**	**OJ**	**C**	**OJ**
(a)	27.48	6.89	9.56	3.14	7.63	2.21
(b)	56.89	0.51	21.89	0.43	18.77	0.23
(c)	71.39	0.12	39.35	0.18	33.99	0.07
(d)	70.18	0.11	11.51	0.03	10.97	0.03
(e)	82.79	0.00	8.94	0.00	8.78	0.00
(f)	53.43	2.65	22.21	4.58	18.61	1.71
(g)	52.16	2.35	6.21	4.46	5.87	1.57
(h)	65.86	2.25	9.75	2.30	9.28	1.15
(i)	79.65	10.66	11.72	2.45	11.37	2.03
(j)	4.94	1.16	0.73	0.36	0.64	0.28
**Average**	62.20	2.67	15.68	1.79	13.92	0.93
**Standard Deviation**	16.96	3.48	10.50	1.83	8.77	0.90

p.s. The extreme values of Image (j) of crack are excluded in calculation of average and standard deviation.

**Table 3.** Segmentation accuracy of crack (C) and open joint (OJ) segmentations by MSED.

**Image ID**	**Accuracy Index (%)**					

	***Compl***		***Corr***		***Quality***	

	**C**	**OJ**	**C**	**OJ**	**C**	**OJ**
(a)	43.21	50.17	7.60	24.70	6.91	19.84
(b)	49.51	64.02	25.47	30.96	20.22	26.37
(c)	80.29	58.22	28.36	22.44	26.51	19.33
(d)	67.45	97.70	12.57	37.31	11.85	36.98
(e)	76.53	90.89	12.23	57.62	11.79	54.47
(f)	62.14	87.38	22.59	38.87	19.86	36.80
(g)	72.30	88.87	5.11	37.47	5.01	35.79
(h)	83.30	71.50	17.87	15.58	17.25	14.67
(i)	58.20	20.03	18.17	10.63	16.07	7.46
(j)	73.66	62.77	5.12	33.74	5.03	28.11
**Average**	66.66	69.16	15.51	30.93	14.05	27.98
**Standard Deviation**	13.24	23.51	8.33	13.44	7.19	13.60

**Table 4.** Inspected image number in different levels of accuracy indices for pipeline defect detection.

**Percentage (%)**		**< 20**	**40 >∼ ≧ 20**	**60 >∼ ≧ 40**	**80 >∼ ≧ 60**	**≧ 80**

**Index**						
Crack detection using MSED	*Compl*	0	5	12	16	7
	*Corr*	38	2	0	0	0
	*Quality*	39	1	0	0	0
Open joint detection using OTHO	*Compl*	0	1	1	5	33
	*Corr*	6	8	17	8	1
	*Quality*	6	15	11	8	0
